# *Flammulina velutipes* Mycorrhizae Attenuate High Fat Diet-Induced Lipid Disorder, Oxidative Stress and Inflammation in the Liver and Perirenal Adipose Tissue of Mice

**DOI:** 10.3390/nu14183830

**Published:** 2022-09-16

**Authors:** Zhen Luo, Qingying Gao, Yuanfei Li, Yifei Bai, Jing Zhang, Weina Xu, Jianxiong Xu

**Affiliations:** 1 Shanghai Key Laboratory of Veterinary Biotechnology, School of Agriculture and Biology, Shanghai Jiao Tong University, Shanghai 200240, China; 2 Institute of Biological Technology, Nanchang Normal University, Nanchang 330032, China; 3 College of Food Science and Technology, Shanghai Ocean University, Shanghai 201306, China

**Keywords:** *Flammulina velutipes* mycorrhizae, oxidative stress, fatty acids metabolism, liver, perirenal adipose tissue

## Abstract

*Flammulina velutipes* (FV) is edible mushroom that has nutritional and medicinal values. FV mycorrhizae, the by-products of FV, are an abundant source and receive less attention. The objective of this study was to investigate the composition of FV mycorrhizae, and its effects on high fat diet (HFD)-induced lipid disorder, oxidative stress, and inflammatory cytokines, both in the liver and perirenal adipose tissue (PAT) of mice. The results showed that FV mycorrhizae contain abundant trace elements, polysaccharide, amino acids and derivatives, and organic compounds. It was found that 4% FV mycorrhizae (HFDFV) supplementation decreased HFD-induced liver weight and triglyceride (TG) in the plasma, liver and PAT, altered plasma and hepatic fatty acids profiles, promoted gene expression involved in lipid hydrolysis, fatty acid transportation and β-oxidation in the liver and reduced lipid synthesis in the liver and PAT. HFDFV attenuated HFD-induced oxidative stress and pro-inflammatory cytokine by increasing GSH/GSSG, and decreasing levels of MDA and IL6 both in the liver and PAT, while it differentially regulated gene expression of IL1β, IL6, and CCL_2_ in liver and PAT. The results indicated that FV mycorrhizae are effective to attenuate HFD-induced lipid disorder, oxidative stress and inflammation in the liver and PAT, indicating their promising constituents for functional foods and herbal medicine.

## 1. Introduction

Obesity is a major worldwide health challenge. In the past few decades, its prevalence has increased, posing a significant economic burden. Obesity increases the risk of metabolic diseases such as type 2 diabetes mellitus, fatty liver disease and hypertension [1]. The adipose tissues and liver play central roles in lipid metabolism and whole-body energy homeostasis, which is responsible for the fundamental pathogenesis of obesity [2]. Thus, the attenuation of lipid disorders in these organs is an effective way to relieve obesity-related metabolic syndromes [3]. Non-steroidal anti-inflammatory drugs (NSAIDs) are widely used in metabolic disorders and inflammation because of their analgesic, anti-inflammatory and antipyretic effects, but adverse effects also exist, such as drug–drug interactions and organ damage, highlighting the need for and importance of complementary and alternative medicine [4,5,6]. Recently, plant extracts and phytochemicals have attracted extensive attention in the management of weight loss and prevention of metabolic diseases [7].

*Flammulina velutipes* (FV) is an excellent source of bioactive compounds such as vitamins, phenol, fibers, polysaccharides and minerals, and exhibits various biological activities such as anti-oxidative, immunomodulatory, and lowering-cholesterol, which are considered as an alternative strategy for the prevention and treatment of lipid disorders [8,9]. Application of plant-based wastes as functional foods has also received growing interest, with food and agricultural industries generating large quantities of by-products. Furthermore, the stem or mycorrhizae of mushrooms have shown different chemical components and structural characteristics such as polysaccharides conjugation, solubility and antioxidant activities compared with the caps of the mushroom, resulting in differences in their absorption and metabolism [10,11]. Although FV mycorrhizae were able to effectively alleviate HFD-induced lipid metabolism disorders, regulate the gut microbiota and activate the immune function of T lymphocyte [12,13], the chemical composition of FV mycorrhizae, its bioavailability and metabolism, and its effects on the liver and adipose tissue are still lacking. In addition, the perirenal adipose tissues (PAT) play an important role in chronic inflammatory, renal disease and cardiovascular events due to its unique anatomy, physiology and location [14,15]. The effect of FV mycorrhizae on lipid metabolism and inflammation in the PAT is unknown. Thus, this study aimed to explore the composition of FV mycorrhizae, and its effect on HFD-induced lipid disorders, oxidative stress and inflammation, both in the liver and PAT of mice.

## 2. Materials and Methods

### 2.1. Composition Analysis

The major compositions, including crude protein (CP), ethanol extract (EE), crude fiber (CF), Ash, Ca and P, harmful substances such as aflatoxin B1 (AFB1), zearalenone (ZEN), deoxynivalenol (DON) and Salmonella, were determined as described previously [16]. The heavy metals (Pb, Cd and Hg) and trace elements (Fe, Mn, Cu, Zn, Se, Na, Mg and K) in FV mycorrhizae were determined using inductively coupled plasma mass spectrometry in Centre Testing International (Qingdao) Co., Ltd. An untargeted and targeted metabolomics approach based on ultrahigh-performance liquid chromatography and equipped with quadrupole time-of-flight mass spectrometry (UHPLC-QTOF-MS) and multiple reaction monitoring (MRM) was used to analyze the composition and quantify the contents of amino acids and derivatives in FV mycorrhizae, as we described previously [17].

### 2.2. Preparation of Polysaccharide

Polysaccharides from dried FV mycorrhizae were extracted according to the water-extraction and alcohol-precipitation method. Briefly, the dried sample were crushed and dissolved in ethanol solution overnight, then extracted by hot water in sample:water = 1:10 for 4 h at 60 °C. The precipitate was re-extracted twice and the supernatants were collected and pooled. Then, the solution was concentrated and precipitated with four volumes of ethanol at 4 °C to obtain crude water-extracts. The extracts were re-dissolved in water, deproteinized, dialyzed, concentrated and freeze dried to obtain crude water polysaccharide. The purity of crude polysaccharide (total soluble sugar) was determined by the sulfuric acid-anthrone method. Total sugar content mg/mg = C*V/M, where C is the calculated result according to the standard curve; M is the actual weighing mass; V is the sample dissolved volume.

### 2.3. Analysis of Monosaccharide Compositions

The monosaccharide compositions were determined according to previous study [18]. Briefly, approximately 5 mg of dried polysaccharide was hydrolyzed with 2 M trifluoroacetic acid at 121 °C for 2 h in a sealed tube. The residue was re-dissolved in deionized water and filtered through 0.22 μm microporous filtering film for measurement. Then, the samples were analyzed by high-performance anion-exchange chromatography (HPAEC) on a CarboPac PA-20 anion-exchange column (3 by 150 mm; Dionex) using a pulsed amperometric detector (PAD; Dionex ICS 5000 system, ThermoFisher Scientific, Waltham, MA, USA). The monosaccharide content was calculated according to the calibration curve of monosaccharide standard.

### 2.4. SEC-MALLS-RI Measurement

The samples were dissolved in 0.1M NaNO_3_ aqueous solution at 1 mg/mL concentration and filtered through a filter of 0.45 μm pore size. The samples were dissolved in DMSO solution containing lithium bromide (0.5% *w*/*w*) at 1 mg/mL and filtered through 0.45 μm filter. The homogeneity and molecular weight were measured using SEC-MALLS-RI. The weight and number-average molecular weight (Mw and Mn) and polydispersity index (Mw/Mn) in 0.1 M NaNO_3_ aqueous solution were measured on a DAWN HELEOS-II laser photometer equipped with three tandem columns (300 × 8 mm, Shodex OH-pak SB-805, 804 and 803; Showa Denko K.K., Tokyo, Japan), which was held at 45 °C using a model column heater. Data were acquired and processed using ASTRA6.1.

### 2.5. Fourier-Transform Infrared Spectra (FT-IR)

The dried polysaccharides were mixed with potassium bromide powder and then pressed into 1 mm pellets for FT-IR measurement (Nicolet iZ-10, ThermoFisher Scientific, Waltham, MA, USA) in the range of 400 to 4000 cm^−1^.

### 2.6. Animal Experiments

Twenty-seven 8-week-old C57BL/6J mice were obtained from Huafukang Bioscience (Beijing, China). The mice were housed in box cages at 22–25 °C under 12 h light/night cycles with ad libitum access to food and water. After 1-week adaptation, they were randomly divided into three groups: control (CON), high fat diet (HFD) and HFD supplemented with +4% FV mycorrhizae powder (HFDFV). Mice that received basic diet served as CON. The CON group (12% fat, #D12450B) and HFD group (60% fat, #D12492) were provided by Huafukang Bioscience (Beijing, China). The HFDFV was evenly mixed HFD with 4% FV mycorrhizae powder, and the blend were extruded to cylinder manually. The experiment lasted for 16 weeks. Body weight and food intake were recorded every 3 days. At the end of the experimental period, the mice were sacrificed followed by exsanguination. The blood samples were collected. Then, livers, PAT and epididymal adipose tissues (EAT) were weighted, washed with PBS, sectioned and stored at −80 °C. The colon length was measured. Fresh tissues were also immediately fixed in 4% paraformaldehyde for further analysis.

### 2.7. Histopathological Examination

The fresh livers were fixed in 4% paraformaldehyde and embedded in paraffin, sectioned and stained with hematoxylin and eosin (H&E) for histopathologic analysis. The liver samples were also embedded in the frozen liver section for oil red staining to visualize lipid droplets.

### 2.8. Triglyceride (TG), Cholesterol (TC) and Transaminase Determination

The plasma was collected after centrifuging at 3500× *g* for 20 min. The liver and PAT tissues were homogenized in PBS buffer and the supernatants were gathered after centrifuging 5000× *g* for 10 min. Protein concentration was measured through the bicinchoninic acid (BCA) protein assay kit. The content of TG, TC and activity of alanine aminotransferase (ALT) in these samples were determined according to the manufacturers’ instructions (Nanjing Jiancheng Bioengineering Institute, Nanjing, China), as in our previous description [19].

### 2.9. Oxidative Stress Parameters Determination

The oxidative stress parameters were determined according to the manufacturers’ instructions (Nanjing Jiancheng Bioengineering Institute, Nanjing, China), as in our previous description [20]. Protein concentration was measured through the bicinchoninic acid (BCA) protein assay kit. Superoxide dismutase (SOD), malondialdehyde (MDA), H_2_O_2_, GSH and GSSG were measured and the absorbance at 450, 530, 405, 405 and 405 nm was recorded, respectively.

### 2.10. Cytokines Determination

The cytokines such as IL1β, IL6, TNFα and CCL_2_ in the liver and PAT tissues were determined using commercially available enzyme-linked immune sorbent assay (ELISA) kits (Nanjing Jiancheng Bioengineering Institute, Nanjing, China), as in our previous description [20]. Briefly, liver and PAT tissues were homogenized in PBS and then centrifuged at 12,000× *g* for 15 min. The microplates were coated with IL1β, IL6, TNFα and CCL_2_, followed by detection with a horseradish peroxidase-labeled substrate after incubation for 10 min at 37 °C. Absorbance values were read in a spectrophotometer at 450 nm.

### 2.11. Untargeted Metabolomics

An untargeted metabolomics approach was used to study the changes in plasma metabolites among three dietary treatments. Analyses were performed using UHPLC (Agilent 1290 Infinity LC) coupled to a QTOF (AB Triple TOF 6600) in Shanghai Applied Protein Technology (APT, Shanghai). Plasma samples were mixed with pre-cooling methanol/acetonitrile/water solution (2:2:1, *v*/*v*). The mixtures were vortexed using an ultrasonator for 30 min and placed at −20 °C for 10 min, centrifuged at 14,000× *g* for 20 min. The vacuum dried supernatants were dissolved in 100 µL acetonitrile solution (acetonitrile:water= 1:1, *v*/*v*) for further analysis. The determination methods and process were carried out according to our previous description [17].

### 2.12. Targeted Metabolomics

A targeted metabolomics approach was used to study the compositions of medium long chain fatty acids in the liver between HFDFV and HFD. Briefly, 10% H_2_SO_4_-CH_3_OH solution (600 µL) were added into the 50 mg sample, suspended for 1 min and bathed in water at 62 °C for 2 h. After cooling, anhydrous sodium sulfate and 600 µL n-hexane were added and suspended for 1 min. Then, the solution was centrifuged at 3500 r/min for 5 min. The supernatants were dried vacuum, dissolved in 200 μL n-hexane and analyzed by GC-MS. The samples were separated by gas chromatography on a DB-5MS capillary column (30 m × 0.25 mm ID × 0.25 μm). A standard sample mixture was used to identify the fatty acids profiles. The concentrations were calculated based on the chromatogram peak areas.

### 2.13. RNA Isolation, cDNA Synthesis and Real-Time PCR

The total RNA from liver and PAT tissues was extracted following the instructions of the RNA extraction kit. The concentration of RNA was quantified using a spectrophotometer (ThermoFisher Scientific, Waltham, MA, USA). Then, 1 μg of RNA was reverse transcribed into cDNA using the PrimeScrip RT reagent Kit. The real-time quantitative PCR reaction was applied to quantify the gene expression with the LightCycler96 system (Roche). The reaction was performed in a total volume of 20 μL, including 10 μL SYBR Green mix, 2 μL cDNA, 7.2 μL H_2_O, and 0.4 μL each of forward and reverse primers. Amplification conditions were initially 95 °C for 30 s, 40 cycles of 95 °C for 5 s and 60 °C for 20 s, and melting curve data. The primers were designed and listed in Supplementary Table S1. β-actin was used as a housekeeping gene to normalize target gene transcript levels. The values were expressed using the formula 2^−(^^△△Ct)^, where △△Ct = (Ct _Target_ − Ct _β-actin_) _treatment_ − (Ct _Target_ − Ct _β-actin_) _HFD_.

### 2.14. Statistical Analysis

Data were analyzed with one-way analysis of variance (ANOVA) followed by post hoc Duncan’s test using the statistical software SPSS 17.0 (SPSS Inc., Chicago, IL, USA), while data of medium long chain fatty acids profiles was analyzed with independent sample *t*-test. Data were presented as mean ± SEM. *p* < 0.05 was considered statistically significant.

## 3. Results

### 3.1. Compositional Analysis of FV Mycorrhizae

**Figure 1:**
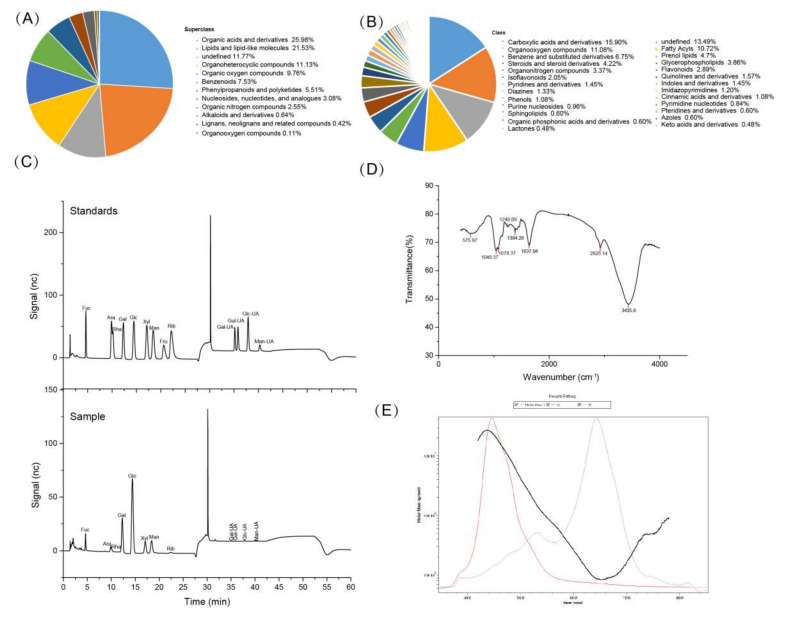
The composition of FV mycorrhizae and characteristics of polysaccharides from FV mycorrhizae were determined. (**A**) Superclass; (**B**) Class; (**C**) The ion chromatography of monosaccharide mixture standards and polysaccharides from FV mycorrhizae; (**D**) The FT-IR spectrum; (**E**) Chromatograms of the molar mass distribution.

The compositions such as CP, EE, CF and ash in FV mycorrhizae are listed in Supplementary Table S2. The levels of AFB1, ZEN and Salmonella were not detected in FV mycorrhizae, and the DON level was very low. The eight essential amino acids (EAAs) Lys, Phe, Met, Thr, Ile, Leu, Val and Trp, non-essential amino acids (NEAAs) such as Tyr, Asp, Asn, Pro, Ala, Ser, Gly, Arg, Glu, Gln, His and cystine were identified (Supplementary Table S3), but the Cys was not detected. The amino acid derivatives such as citrulline, ornithine, hydroxyproline, aminoadipic acid, creatine, creatinine, choline and taurine, biogenic amines such as putrescine were also identified. However, the spermidine was not detected. Untargeted metabolomics showed that 945 compounds (12 superclass and 57 class) were identified (Figure 1A,B), including organic acids and derivatives (25.98%), lipids and lipid-like molecules (21.53%), undefined (11.77%), organoheterocyclic compounds (11.13%), organic oxygen compounds (9.76%), and so on. The specific compositions are listed in the Supplementary Materials.

In total, 1.354 g crude polysaccharide was obtained after extraction and impurity removal from 50 g FV mycorrhizae powder. Thus, the extraction rate was about 2.7%. The purity of polysaccharide was calculated as 52.9% according to the formula. The ion chromatography of monosaccharide mixture standards and polysaccharides from FV mycorrhizae are shown in Figure 1C. They were composed of glucose (Glc), galactose (Gal), mannose (Man), xylose (Xyl), fucose (Fuc), arabinose (Ara), ribose (Rib), rhamnose (Rha), guluronic acid (Gul-UA), mannuronic acid (Man-UA), galacturonic acid (Gal-UA) and glucuronic acid (Glu-UA) in a molar ratio of 46.02:20.67:11.3:7.38:6.95:2.83:1.1:1.09:1.11:1.02:0.32:0.22 (Table 1), indicating that the acidic polysaccharides were rich in glucose. Polysaccharides had three characteristic absorptions peaks, which represented by a strong absorption peak at 3435 cm^−1^ for the stretching vibrations of O-H, absorption peak at 2925.14 cm^−1^ for C-H stretching vibration and absorption peak at 1637.04 cm^−1^ for the stretching vibration of C=O in this study (Figure 1D). Galactose and glucose showed the strongest peak at 1078.37 cm^−1^ and 1040.37 cm^−1^, which is consistent with the monosaccharide composition analysis. The peak at around 1384.28 cm^−1^ and 1248.09 cm^−1^ could be due to the C-O-H of carboxylic acid, S=O or acetyl group (CH3-CO) stretching vibrations, according to a previous study [21]. The molecular mass distribution and chain conformation are shown in Figure 1E. The number-average molar masses (Mw) and weight-average molar masses (Mn) were 1.809 × 10^6^ and 1.632 × 10^5^ g/mol. The Rw, Rn and Rz were 42.6, 45.3 and 37.2 nm (Table 1). The polydispersity value (Mw/Mn) was 11.085, indicating the extract had wide molecular weight distribution.

### 3.2. Effect of FV Mycorrhizae on Mice Body Weight, Food Intake, Organs Weight and Plasma Lipid Disorders

**Figure 2:**
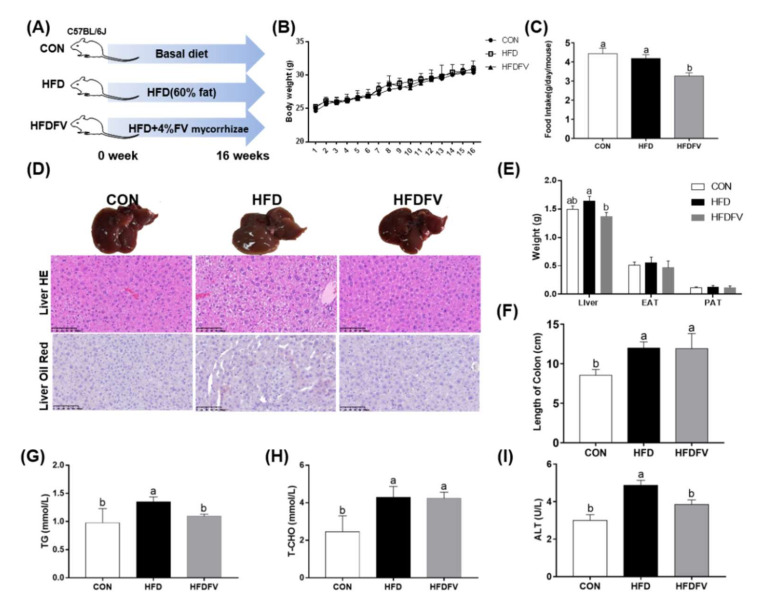
Effect of FV mycorrhizae on the HFD-induced body weight, food intake, liver histopathology and plasma lipids abundance in mice. (**A**) The experimental design. CON: control group, mice were received basic diet. HFD: high fat diet group, mice were received 60% high fat diet. HFDFV: high fat diet + 4% FV mycorrhizae group, mice were received 60% high fat diet supplemented with 4% FV mycorrhizae powder; (**B**) The body weight; (**C**) Food intake; (**D**) The liver morphology, HE and oil red staining; (**E**) The weight of liver, PAT and EAT; (**F**) The length of colon; (**G**) The content of plasma TG; (**H**) The content of plasma TC; (**I**) The activity of plasma ALT. C57BL/6J mice were divided into 3 groups and were fed either basal diet (CON, 10% fat), high-fat diet (HFD, 60% fat) or HFD + 4% FV (HFDFV) for 16 weeks. Values with different letters differ significantly (*p* < 0.05). Values were expressed as mean ± SEM (*n* = 6–8).

As shown in Figure 2B, the mice body weight was not significantly different (*p* > 0.05) among the three groups from the beginning to the end of the experimental period. Compared with the CON, HFD had no effect on food intake (*p* > 0.05), while HFDFV significantly decreased food intake compared with the HFD (*p* < 0.05) (Figure 2C). Liver HE and oil red staining are shown in Figure 2D. HFDFV improved HFD-induced structural damage in liver by HE staining. The weight of PAT and EAT were not affected by dietary treatment (*p* > 0.05) and the weight of liver was significantly decreased by 16.5% in HFDFV compared with HFD (*p* < 0.05) (Figure 2E). HFD significantly increased the length of the colon compared with CON (*p* < 0.05), but HFDFV had no effect on the length of the colon compared with HFD (*p* > 0.05) (Figure 2F). HFDFV significantly decreased the HFD-induced content of plasma TG (*p* < 0.05) (Figure 2G) but had no effect on the content of plasma TC (*p* > 0.05) (Figure 2H). Additionally, HFDFV significantly decreased the HFD-induced activity of ALT in plasma (*p* < 0.05) (Figure 2I). These results indicated that FV mycorrhizae treatment reduced HFD-induced lipid disorder and liver dysfunction in mice.

### 3.3. Effect of FV Mycorrhizae on Lipid Metabolism and Oxidative Stress Parameters in the Liver and PAT

**Figure 3:**
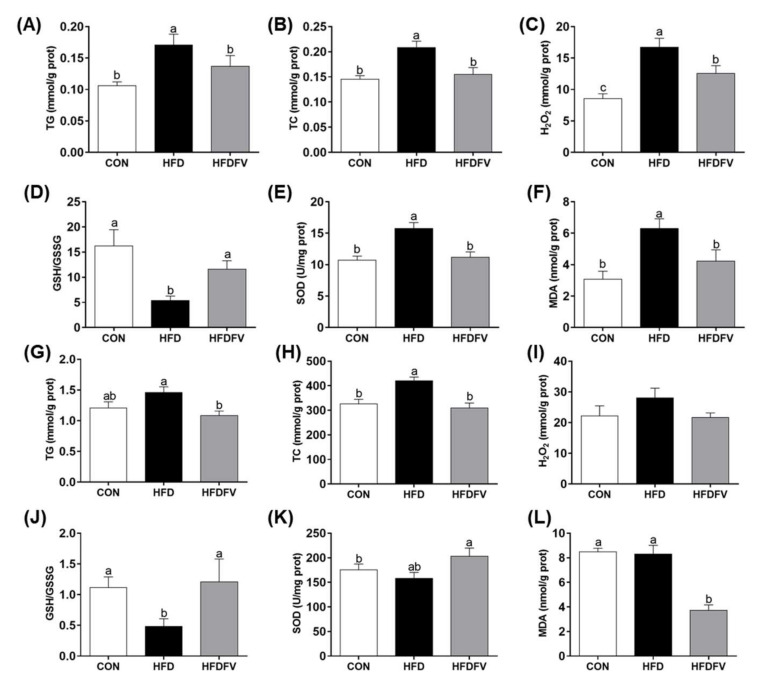
Effect of FV mycorrhizae on the lipids content and oxidative stress parameters in the liver and PAT of mice. (**A**–**F**) Lipid content and oxidative stress parameters in the liver; (**G**–**L**) Lipid content and oxidative stress parameters in the PAT. TG, triglyceride; TC, total cholesterol. Values with different letters differ significantly (p < 0.05). Values were expressed as mean ± SEM (n = 6–8).

The lipid metabolism and oxidative stress parameters in the liver and PAT were further studied. In the liver, HFDFV significantly decreased the HFD-induced content of TG and TC (*p* < 0.05) (Figure 3A,B). Oxidative stress parameters showed that HFDFV significantly decreased the HFD-induced content of H_2_O_2_, MDA and the activity of SOD, increased GSH/GSSG in the liver (*p* < 0.05) (Figure 3C–F). In the PAT, HFDFV significantly decreased the HFD-induced content of TG and TC (*p* < 0.05) (Figure 3G,H). Oxidative stress parameters showed that HFDFV significantly decreased the HFD-induced content of MDA and increased the activity of SOD and GSH/GSSG in the PAT (*p* < 0.05). The content of H_2_O_2_ in the PAT was not affected by dietary treatments (*p* > 0.05) (Figure 3I–L). These results indicated that FV mycorrhizae reduced HFD-induced fat accumulation and oxidative injury and improved anti-oxidative capacity in the liver and PAT of mice.

### 3.4. Effect of FV Mycorrhizae on Gene Expression of Lipid Metabolism and Mitochondrial Biogenesis in the Liver and PAT

**Figure 4:**
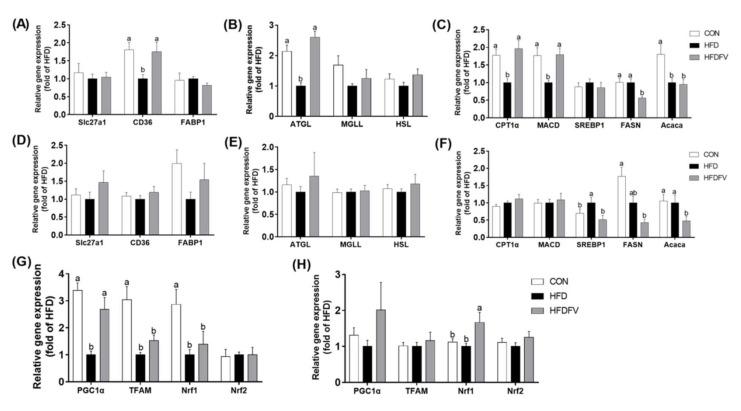
Effects of FV mycorrhizae on HFD-induced gene expression of lipid transport (slc27a1, CD36 and FABP1), lipases (ATGL, MGLL and HSL), β-oxidation (CPT1a and MACD), synthesis (FASN, Acaca and SREBP1) and mitochondrial biogenesis in the liver and PAT of mice. (**A**) Gene expression of lipid transport in the liver; (**B**) Gene expression of lipases in the liver; (**C**) Gene expression of β-oxidation and synthesis in the liver; (**D**) Gene expression of lipid transport in the PAT; (**E**) Gene expression of lipases in the PAT; (**F**) Gene expression of β-oxidation and synthesis in the PAT. (**G**) Gene expression of mitochondrial biogenesis in the liver; (**H**) Gene expression of mitochondrial biogenesis in the PAT. Values with different letters differ significantly (p < 0.05). Values were expressed as mean ± SEM (n = 6–8).

The gene expression of fatty acids transport (CD36, Slc27a1 and FABP1), hydrolysis (ATGL, HSL and MGLL), β-oxidation (CPT1α and MACD) and synthesis (SREBP1, FASN and Acaca) in the liver and PAT were further studied (Figure 4). In the liver, compared with the CON, HFD significantly decreased the gene expression of CD36, ATGL, CPT1α, MACD and Acaca, while HFDFV significantly increased the gene expression of CD36, ATGL, CPT1α and MACD, decreased the expression of FASN in the liver (*p* < 0.05) (Figure 4A–C). The gene expression of Slc27a1, FABP1, MGLL, HSL and SREBP1 in the liver were not affected by dietary treatment (*p* > 0.05). In the PAT, compared with the CON, HFD had no effect on the gene expression of Slc27a1, CD36, FABP1, ATGL, MGLL, HSL, CPT1α and MACD (*p* > 0.05). Compared with the HFD group, HFDFV significantly decreased the gene expression of SREBP1, FASN and Acaca in the PAT (*p* < 0.05) (Figure 4D–F). These results suggested that FV promoted fatty acids transport, hydrolysis and β-oxidation in the liver and inhibited fatty acids synthesis both in the liver and PAT.

Compared with the CON, HFD significantly decreased the gene expression of mitochondrial biogenesis PGC1α, TFAM and Nrf1 (*p* < 0.05), but did not affect Nrf2 in the liver (*p* > 0.05). HFDFV significantly increased HFD-induced gene expression of PGC1α in the liver (*p* < 0.05) and had no effect on the gene expression of TFAM, Nrf1 and Nrf2 (*p* > 0.05) (Figure 4G). In the PAT, compared with the CON, HFD had no effect on the gene expression of PGC1α, TFAM, Nrf1 and Nrf2 (*p* > 0.05). HFDFV significantly increased the HFD-induced gene expression of Nrf1 in the PAT (*p* < 0.05) and had no effect on the gene expression of PGC1α, TFAM and Nrf2 in the PAT (*p* > 0.05) (Figure 4H).

### 3.5. Effect of FV Mycorrhizae on Inflammation in the Liver and PAT

**Figure 5:**
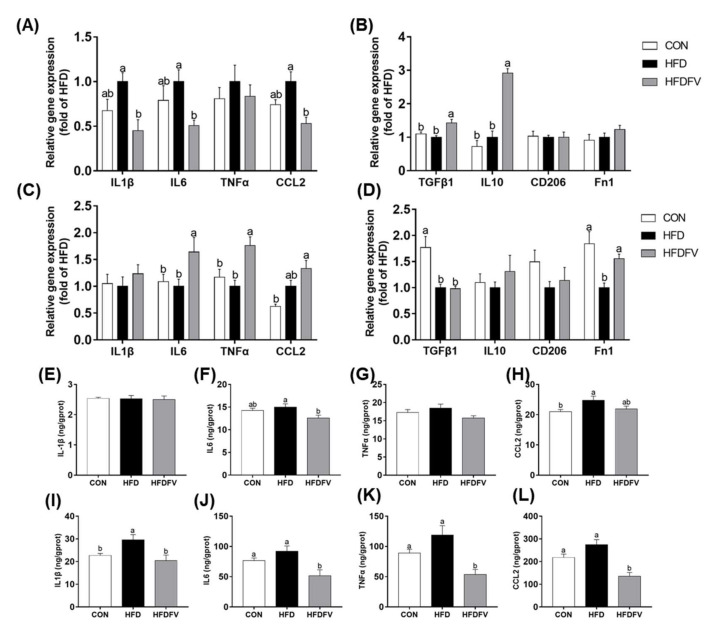
Effects of FV mycorrhizae on HFD-induced inflammatory cytokines in the liver and PAT of mice. (**A**) Gene expression of pro-inflammatory cytokines in the liver; (**B**) Gene expression of anti-inflammatory cytokines in the liver; (**C**) Gene expression of pro-inflammatory cytokines in the PAT; (**D**) Gene expression of anti-inflammatory cytokines in the PAT; (**E**–**H**) The contents of pro-inflammatory cytokines in the liver; (**I**–**L**) The contents of pro-inflammatory cytokines in the PAT. Values with different letters differ significantly (*p* < 0.05). Values were expressed as mean ± SEM (*n* = 6–8).

The pro-inflammatory M1 markers (IL1β, IL6, TNFα and CCL_2_) and anti-inflammatory M2 markers (TGFβ1, Fn1, CD206 and IL10) were further studied in the liver and PAT. Compared with the CON, HFD had no significant effect on the gene expression of IL1β, IL6, TNFα and CCL_2_ in the liver (*p* > 0.05). Compared with HFD, HFDFV significantly decreased the gene expression of IL1β, IL6 and CCL_2_ in the liver (*p* < 0.05) and had no effect on the gene expression of TNFα in the liver (*p* > 0.05) (Figure 5A). Compared with the CON, HFD had no effect on the gene expression of TGF-β1, Fn1, CD206 and IL10 in the liver (*p* > 0.05), whereas HFDFV significantly increased the gene expression of TGFβ1 and IL10 in the liver (*p* < 0.05) (Figure 5B). In the PAT, compared with the CON, HFD had no effect on the gene expression of IL1β, IL6, TNFα and CCL_2_ (*p* > 0.05). Compared with HFD, HFDFV significantly increased the gene expression of IL6 and TNFα in the PAT (*p* < 0.05) and had no effect on the gene expression of IL1β and CCL_2_ in the PAT (*p* > 0.05) (Figure 5C). HFDFV had no effect on the gene expression of TGFβ1, IL10 and CD206 in the PAT (*p* > 0.05), but significantly increased the gene expression of Fn1 in the PAT (*p* < 0.05) (Figure 5D).

The contents of pro-inflammatory cytokines in the liver and PAT were further studied by ELISA. Compared with the CON, HFD increased CCL_2_ content in the liver (*p* < 0.05), had no effect on the contents of IL1β, IL6 and TNFα (*p* > 0.05). HFDFV significantly decreased the content of IL6 in the liver compared with HFD (*p* < 0.05) and had no effect on the contents of IL1β, IL6 and CCL_2_ in the liver (*p* > 0.05) (Figure 5E–H). In the PAT, HFDFV significantly decreased the content of IL1β, IL6, TNFα and CCL_2_ in the PAT compared with HFD (*p* < 0.05) (Figure 5I–L).

### 3.6. Effect of FV Mycorrhizae on Differential Metabolites in Plasma

**Figure 6:**
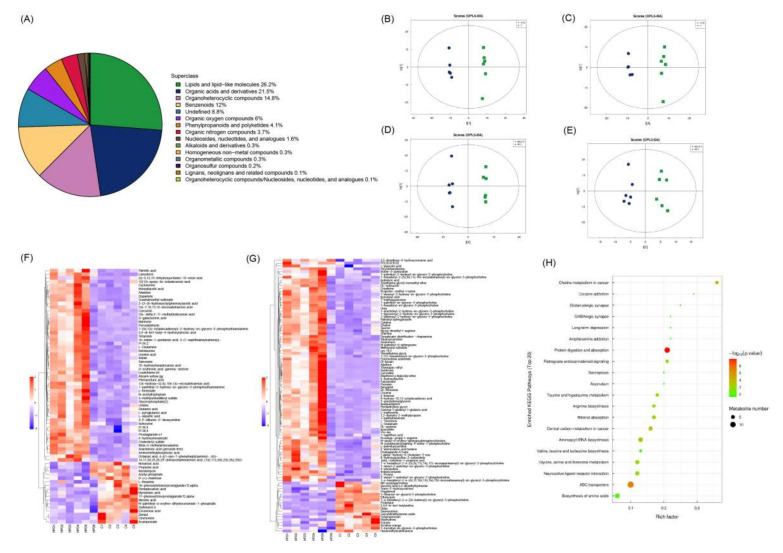
The differential metabolites were determined by untargeted metabolomics. (**A**) The classification of identified metabolites; OPLS-DA analysis HFD vs. CON (**B**,**C**) and HFDFV vs. HFD (**D**,**E**) under negative and positive ions model; (**F**,**G**) Hierarchical clustering of differential metabolites in HFD vs. CON under negative and positive ions, respectively; (**H**) KEGG enrichment analysis of HFD vs. CON.

The untargeted metabolomics method was used to identify plasma differential metabolites among three groups. In total, 1000 metabolites were ultimately obtained including lipids and lipid-like molecules (26.2%), organic acids and derivatives (21.5%), organoheterocyclic compounds (14.8%), benzenoids (12%), undefined (8.8%), organic oxygen compounds (6%), phenylpropanoids and polyketides (4.1%), organic nitrogen compounds (3.7%), nucleosides, nucleotides, and analogues (1.6%), alkaloids and derivatives (0.3%) and so on (Figure 6A). PCA with 7-fold cross-validation showed the model interpretation rates were R2X = 0.582 and 0.54 (HFD vs. CON) and 0.545 and 0.536 (HFDFV vs. HFD) under negative and positive ions, respectively. OPLS-DA with 7-fold cross-validation showed Q2 = 0.733 and 0.66 (HFD and CON) and 0.389 and 0.384 (HFDFV vs. HFD) under negative and positive ions (Figure 6B–E). There was no overfitting in the permutation test under the negative and positive ions model. These results indicate that dietary treatment significantly changed the plasma metabolites. A VIP > 1 and *p* < 0.05 were used as criteria for differential metabolite screening.

Compared with the CON, 167 differential metabolites were identified in HFD under the negative and positive ions model (Figure 6F,G and Table 2). Compared with CON, the plasma fatty acids such as palmitic acid (C16:0) and 16-hydroxyhexadecanoic acid, unsaturated fatty acids (UFA) such as linoleic acid (C18:2), arachidonic acid (C20:4), 14,17,20,23,26,29-dotriacontahexaenoic acid (14z,17z,20z,23z,26z,29z)-, Cis-7,10,13,16-docosatetraenoic acid, Cis-.delta.2-11-methyldodecenoic acid, 9-hydroxy-10,12-octadecadienoic acid, 12s-hydroxy-5z,8z,10e,14z-eicosatetraenoic acid, amino acids such as Gln, Glu, Asn, Ile, taurine, Thr, Met, Arg and Pro were increased, while myristic acid (C14:0), myristoleic acid, pentadecanoic acid (C15:0) and eicosenoic acid (C20:1) decreased in the HFD group, indicating that HFD induced plasma fatty acid disorders and increased levels of amino acids in mice. KEGG pathways showed that HFD affected pathways involving protein digestion and absorption, ABC transporters, aminoacyl-tRNA biosynthesis, central carbon metabolism in cancer and so on (Figure 6H). Compared with the HFD group, 39 differential metabolites under the negative and positive ion model were identified in the HFDFV group (Table 2). HFDFV decreased HFD-induced plasma fatty acids such as linoleic acid (C18:2) and cis-.delta.2-11-methyldodecenoic acid, suggesting that FV mycorrhizae supplementation can effectively inhibit HFD-induced fatty acid disorders in mice. However, there were no KEGG enrichment pathways between HFDFV and HFD in the present study.

### 3.7. Effect of FV Mycorrhizae on Medium Long Chain Fatty Acids in the Liver

Furthermore, the medium long chain fatty acids including saturated fatty acids (SFA), trans fatty acids (TFA), monounsaturated fatty acids (MUFA) and polyunsaturated fatty acids (PUFA) in the liver between HFD and HFDFV groups were determined by targeted metabolomics (Table 3). Compared with the HFD group, HFDFV significantly increased the contents of lauric acid (C12:0), TFA such as linoelaidic acid (C18:2TT), nervonic acid (C24:1) and docosapentaenoate (C22:5*n*-3) in the liver (*p* < 0.05). The contents of 11C,14C-eicosadienoic acid (C20:2), docosapentaenoate (C22:5*n*-6) and adrenic acid (C22:4) were decreased in HFDFV compared with HFD (*p* < 0.05). Other fatty acids were not significantly different between the two groups (*p* > 0.05).

## 4. Discussion

Lipid metabolic disorder is one of the important factors that cause obesity. Liver and adipose tissue are the major sites responsible for lipid metabolism, transportation and storing. In this study, although the body weight of mice was not significantly different among treatments, the results showed that HFDFV decreased HFD-induced liver weight, plasma TG and ALT, TG and TC in liver and PAT. Furthermore, metabolomics results showed that HFD increased the plasma palmitic acid (C16:0) and 16-hydroxyhexadecanoic acid, linoleic acid (C18:2) and arachidonic acid (C20:4), decreased myristic acid (C14:0), pentadecanoic acid (C15:0) and eicosenoic acid (C20:1), while HFDFV reversed HFD-induced plasma linoleic acid (C18:2) and cis-.delta.2-11-methyldodecenoic acid. SFA such as palmitic acid is abundant in human serum and western-style diet, and appeared more powerful than UFA at inducing metabolic disorders [22,23]. A high level of 16-hydroxyhexadecanoic acid was related to chronic kidney disease [24]. High intake of essential fatty acids linoleic acid (C18:2) and its derivative arachidonic acid (C20:4) induced inflammation, adipogenesis and lipid accumulation in male mice, although the role of linoleic acid in obesity development is still contentious [25,26]. Myristic acid (C14:0) and odd chain fatty acid pentadecanoic acid (C15:0) are negatively associated with liver fat, injury and non-alcoholic steatohepatitis [27,28]. These studies suggested that FV mycorrhizae mainly inhibited HFD-induced lipid disorders through decreasing plasma UFA in mice. Meanwhile, HFDFV increased the contents of lauric acid (C12:0), linoelaidic acid (C18:2TT), nervonic acid (C24:1) and docosapentaenoate (C22:5*n*-3) in the liver, decreased 11C,14C-eicosadienoic acid (C20:2), docosapentaenoate (C22:5*n*-6) and adrenic acid (C22:4). Lauric acid is rich in coconut oil and has a anti-inflammation, antibacterial activity and anti-obesity role [29,30]. Nervonic acid was reported to negatively correlate with obesity; its supplementation could effectively treat obesity through increasing fatty acids β-oxidation [31,32]. Docosapentaenoate (C22:5*n*-3) is an intermediate product between eicosapentaenoic acid (EPA) and docosapentaenoic acid (DHA) that exhibit good efficacy in lowering lipids as well as their isomer docosapentaenoate (C22:5*n*-6) [33]. Adrenic acid is an important factor to promote the disease progression of NAFLD through the induction of inflammation [34]. Indeed, present results showed that HFDFV inhibited pro-inflammatory gene expression (IL1β, IL6 and CCL_2_) and increased anti-inflammatory gene expression (TGFβ and IL10) in liver. Thus, these studies suggested that FV mycorrhizae was effective to improve metabolic disorders and decrease plasma UFA, possibly by promoting fatty acids β-oxidation and inhibiting inflammation in liver.

Slc27a1, CD36 and FABP1 are required for fatty acid uptake and transport [35]. The decrease in gene expression of CD36 in liver in this study indicated a restriction in fat mass gain when fed a high fat diet. Metabolic lipases such as ATGL, HSL and MGLL are responsible for lipid catabolism by hydrolyzing TG into diacylglycerols (DAG), monoacylglycerols (MAGs) and free FAs and glycerol, respectively [36]. CPT1α is the rate-limiting enzyme of fatty acid β-oxidation involved in mitochondrial fatty acid uptake [37]. MACD regulates the first step of fatty acid β-oxidation by converting acyl-coenzyme A into trans-enoyl-CoA [38]. Acaca is the rate-limiting enzyme of de novo fatty acid biosynthesis that catalyzes the carboxylation of acetyl-CoA to malonyl-CoA [39]. FASN is a cytosolic metabolic enzyme that catalyzes de novo fatty acid synthesis, which is transcriptionally regulated by SREBP1 [40]. In this study, FV increased HFD-induced gene expression of CD36, ATGL, CPT1α and MACD, decreased the gene expression of FASN in the liver and inhibited the gene expression of SREBP1, FASN and Acaca in the PAT, suggesting that FV mycorrhizae effectively inhibited lipid accumulation by promoting fatty acid transportation, lipid hydrolysis and β-oxidation in the liver and reducing lipid synthesis in the liver and PAT. These were similar to previous studies, which showed that FV polysaccharide, powder and mixture attenuated HFD-induced obesity and hyperlipidemia through increasing oxidation and decreasing synthesis in the liver and PAT [13,41,42,43].

Excessive FFA intake also induces lipotoxicity, resulting in increased oxidative stress and inflammation [44]. SOD is responsible for preventing O_2_^−^-induced oxidative damage. MDA is the final products of PUFA peroxidation indicating lipid peroxidation [45]. The decreased ratio of GSH/GSSG indicated an impairment of redox status in the cell [46]. In this study, HFDFV reversed the HFD-induced activities of SOD and the content of H_2_O_2_, MDA and GSH/GSSG in the liver and MDA and GSH/GSSG in the PAT, suggesting that FV mycorrhizae attenuated HFD-induced oxidative stress, and improved anti-oxidative ability in the liver and PAT. Obesity was also a kind of low-grade chronic inflammatory response, with increasing circulating levels of inflammatory cytokines, such as TNFα and IL6. PAT was active to secrete adipokines and cytokines compared with other visceral adipose tissue, playing an important role in renal metabolism and cardiovascular system by paracrine or endocrine pathways [47]. In this study, HFDFV decreased the contents of IL1β, IL6, TNFα and CCL_2_ in the PAT, and IL6 content in the liver, suggesting FV mycorrhizae inhibited HFD-induced inflammation, mainly through inhibiting the secretion of pro-inflammatory cytokines from PAT. Of note, HFDFV increased the gene expression of IL6, TNFα in the PAT, which is contrary to the liver. In fact, the mushroom extracts were reported to differentially regulate immune cells such as T-lymphocytes, monocytes, NK and T cells and cytokines secretion dependent on mushroom compound compositions, extract methods, absorption and bioavailability, synergistic or antagonistic effects [48]. On the other hand, cytokines such as TNFα were also reported to drive lipolysis. The adipose tissues-liver crosstalk is responsible for lipid metabolic balance, but it is also sensitive to dietary types, composition and amount [49]. Thus, the specific ingredients and mechanism responsible for FV mycorrhizae regulating host immune response between adipose tissue and liver remain unknown and need further investigation. Of note, HFDFV increased plasma gingerols and probucol and decreased coumarin compared with the HFD group. 6-gingerols are the major pungent phenolic compounds present in the rhizomes of ginger, which showed antioxidant, anti-inflammatory, and antihyperglycemic activity [50]. Probucol is a well-known natural product antioxidant that can effectively treat hyperlipidaemia and NAFLD [51]. Coumarin is a plant-derived natural compound that showed antioxidant, antibacterial and anti-inflammation ability. These results suggest that the anti-oxidative and hypolipidemic ability of FV mycorrhizae possibly attributed to these antioxidants. However, these ingredients were not found in the composition of FV mycorrhizae in our results, indicating the metabolism and transform of mycorrhizae or their low contents under detection, which need further verification.

## 5. Conclusions

In this study, the results showed that FV mycorrhizae contained multifarious nutritive components, including abundant trace elements, polysaccharide, amino acids and derivatives, and organic small molecule compounds, which attenuated HFD-induced lipid disorders in the plasma and liver, inhibited oxidative stress and differentially regulated inflammation in the liver and PAT of mice, indicating the application of promising constituents of FV mycorrhizae for functional foods and herbal medicine.

## Figures and Tables

**Table 1:** Characteristics of polysaccharides extracted from FV mycorrhizae.

Item	Results
Sugar composition (%)	
Glc	46.02
Gal	20.67
Man	11.3
Xyl	7.38
Fuc	6.95
Ara	2.83
Rib	1.1
Rha	1.09
Fru	0
Gul-UA	1.11
Man-UA	1.02
Gal-UA	0.32
Glu-UA	0.22
Molecular characteristics	
Mn (g/mol)	1.632 × 10^5^
Mw (g/mol)	1.809 × 10^6^
Mz (g/mol)	1.427 × 10^7^
Polydispersity (Mw/Mn)	11.085
Rn (nm)	45.3
Rw (nm)	42.6
Rz (nm)	37.2

**Table 2:** Identification of differential metabolites between HFDFV and HFD under negative and positive ionization mode.

Adduct	Name	VIP	FC	*p*-Value	*m*/*z*
Negative					
[M-H]-	[6]-gingerol	5.634407	1.422392	0.000207	293.1763
[M-H-C_6_H_12_O]-	8,11-tridecadienoic acid, 13-(3-pentyl-2-oxiranyl)-, (8z,11z)-	1.649861	1.3444	0.00024	221.1545
[M-H]-	Tetradec-5-ynoic acid	1.299375	0.357147	0.00122	223.1702
[M-H-C_17_H_27_SO]-	Probucol	2.159909	1.65893	0.003397	236.1054
[M-H]-	Cis-.delta.2-11-methyldodecenoic acid	1.599766	0.186622	0.004097	211.1701
[M-H]-	12-hydroxydodecanoic acid	1.252276	1.765689	0.006782	215.1654
[M-H-CO_2_]-	3,5-di-tert-butyl-4-hydroxybenzoic acid	1.876308	0.365134	0.021764	205.1596
[M-H-C_18_H_34_O_2_]-	1,2-dioleoyl-sn-glycero-3-phosphate	1.975814	2.621357	0.022762	417.2414
[M-H]-	1h-indole-1-pentanoic acid, 3-(1-naphthalenylcarbonyl)-	3.057796	0.318523	0.023438	370.1697
[M+Cl]-	1-palmitoyl-2-arachidonoyl-sn-glycero-3-phosphocholine	1.347016	0.665036	0.031371	816.5336
[M-H]-	Linoleic acid	14.5538	0.618988	0.033811	279.2333
[M-H]-	2′,2′-difluoro-2′-deoxyuridine	2.68574	0.656394	0.03705	263.0445
[M-H]-	Deoxythymidine 5′-phosphate (dTMP)	2.468689	3.258614	0.040277	321.0441
[M-H]-	3-hydroxycapric acid	1.544758	1.462081	0.041071	187.1337
Positive					
[M+H]+	Lauryldimethylamine oxide	4.245431	0.489801	0.000229	230.2479
[M+H]+	N-acetyl-o-fluoro-dl-phenylalanine	3.723481	1.206781	0.003209	226.084
[M+H]+	Coumarin	1.934626	0.525499	0.00427	147.0554
[M+H-NH_3_]+	Porphobilinogen	3.582239	1.384773	0.005902	210.0796
(M+H)+	L-Proline	1.783547	0.545155	0.005949	116.0709
[M+Na]+	Stavudine	1.570389	1.749773	0.009195	247.0607
[M+H-H_2_O]+	Prostaglandin e3	1.374113	0.454302	0.012679	333.2059
[M+H]+	Pro-Trp	1.402203	0.572081	0.01372	302.3052
[M+H]+	Tetraethylene glycol monomethyl ether	5.241345	0.431367	0.013853	209.1385
[M+H]+	Fenpropimorph	1.4829	1.619531	0.014177	304.2845
[M+H]+	Pentapropylene glycol	2.740352	0.51482	0.016788	309.227
[M+H-H_2_O]+	Fingolimod	1.43912	0.595578	0.016981	290.2688
[M+H]+	3,4-dimethylmethcathinone	1.876348	0.319043	0.020052	192.1596
[M+H]+	Triethylene glycol monobutyl ether	3.491736	1.698806	0.021376	207.1592
[M+H]+	Tributyl phosphate	1.08038	1.705361	0.022941	267.1719
[M+H]+	Decanoyl m-nitroaniline	1.016153	0.557516	0.023725	293.211
(M-H+2Na)+	1-Stearoyl-sn-glycerol 3-phosphocholine	13.17318	1.343167	0.024106	568.3402
[M+H]+	.gamma.-nonalactone	1.413858	1.068891	0.030097	157.1337
[M+H]+	Lpc 18:1	20.32652	1.257069	0.03399	522.3565
[M+NH4]+	Desferrioxamine d2	2.815889	4.933469	0.034191	604.3541
[M+NH4]+	1,2-dilinoleoylglycerol	1.123168	0.244778	0.036202	634.5408
[M+H]+	2-fluoroamphetamine	1.286169	1.631088	0.039465	154.0863
[M+H-CH_2_O_2_]+	4-hydroxynonenal alkyne	1.267965	3.974206	0.042399	107.0859
[M+Na]+	Anisomycin	1.322298	3.083059	0.04541	288.144
[M+H]+	Fenpropidin	9.809554	0.31689	0.049266	274.2742

**Table 3:** Effects of FV mycorrhizae on HFD-induced medium long chain fatty acids profiles (mg/g Tissue) in the liver.

Item		HFD	HFDFV	*p*
SFA		2973.287 ± 356.423	2865.914 ± 262.981	0.813
Octanoic acid	C8:0	0.259 ± 0.033	0.22 ± 0.019	0.335
Decanoic acid	C10:0	0.489 ± 0.095	0.468 ± 0.039	0.838
Lauric acid	C12:0	0.247 ± 0.018	0.337 ± 0.036	0.047
Tridecanoic acid	C13:0	0.368 ± 0.026	0.361 ± 0.018	0.827
Myristic acid	C14:0	3.577 ± 0.551	5.19 ± 0.922	0.164
Pentadecanoic Acid	C15:0	1.907 ± 0.225	2.522 ± 0.178	0.057
Palmitic acid	C16:0	299.472 ± 32.524	308.18 ± 18.933	0.822
Margaric Acid	C17:0	5.693 ± 0.699	6.715 ± 0.698	0.325
Stearic acid	C18:0	2641.364 ± 320.93	2524.592 ± 244.726	0.778
Arachidic Acid	C20:0	5.342 ± 0.661	3.793 ± 0.547	0.101
Docosanoic acid	C22:0	10.2 ± 1.475	8.68 ± 1.272	0.453
Tricosanoic Acid	C23:0	1.262 ± 0.162	1.581 ± 0.17	0.204
Lignoceric Acid	C24:0	3.107 ± 0.526	3.276 ± 0.584	0.834
MUFA		39.561 ± 3.541	40.531 ± 1.791	0.812
Myristelaidic Acid	C14:1	0.608 ± 0.029	0.571 ± 0.038	0.450
10-Pentadecenoic Acid	C15:1	4.307 ± 0.912	4.265 ± 0.632	0.970
Palmitoleic Acid	C16:1	5.177 ± 0.607	5.23 ± 0.36	0.941
Heptadecanoic acid (cis-10)	C17:1	3.704 ± 0.45	4.069 ± 0.321	0.524
Oleic acid	C18:1	0.261 ± 0.109	0.625 ± 0.162	0.092
cis-11-Eicosenoic acid	C20:1	22.144 ± 2.478	20.347 ± 1.561	0.553
Nervonic acid	C24:1	3.36 ± 0.503	5.425 ± 0.629	0.028
TFA		131.714 ± 19.462	208.164 ± 13.972	0.010
Hexadecanoic acid (trans-9)	C16:1T	10.689 ± 2.988	22.393 ± 5.15	0.078
Trans-10-HeptadecenoicAcid(C17:1T)	C17:1T	1.611 ± 0.214	1.944 ± 0.214	0.297
Trans-10-Nonadecenoic acid	C19:1T	2.193 ± 0.307	1.77 ± 0.215	0.286
Trans-11-Eicosenoic Acid	C20:1T	1.653 ± 0.217	1.982 ± 0.262	0.356
Linoelaidic Acid	C18:2TT	115.569 ± 16.333	180.075 ± 11.339	0.009
PUFA		1420.182 ± 231.802	1401.189 ± 153.088	0.947
γ-Linolenic acid	C18:3	14.133 ± 2.116	16.241 ± 1.172	0.404
α-Linolenic acid	C18:3	296.486 ± 38.875	234.847 ± 14.542	0.168
Eicosapentaenoate	C20:5	358.805 ± 40.702	377.603 ± 32.96	0.727
Arachidonic acid	C20:4	443.149 ± 148.283	415.357 ± 134.038	0.892
Cis-11,14,17-Eicosatrienoic Acid	C20:3	54.141 ± 6.682	48.17 ± 5.572	0.508
11C,14C-Eicosadienoic Acid	C20:2	4.505 ± 0.593	2.011 ± 0.379	0.005
Docosapentaenoate (C22:5*n*-6)	C22:5*n*-6	72.843 ± 7.426	39.97 ± 3.493	0.002
Docosapentaenoate (C22:5*n*-3)	C22:5*n*-3	158.709 ± 20.87	254.869 ± 16.861	0.005
Adrenic Acid	C22:4	15.307 ± 1.763	10.138 ± 1.475	0.048
13C,16C-Docosadienoic Acid	C22:2	2.103 ± 0.362	1.985 ± 0.51	0.854

SFA, MUFA, TFA and PUFA were the sum of corresponding fatty acids in this table. Values were expressed as mean ± SEM (*n* = 6).

## Data Availability

Not applicable.

## Supplementary Materials

The following supporting information can be downloaded at: https://www.mdpi.com/article/10.3390/nu14183830/s1, Table S1: Primers used in this study; Table S2: The nutritional composition, harmful substances and heavy metals in FV mycorrhizae; Table S3: Amino acids contents and derivatives in FV mycorrhizae (μmol/g). Reference [52] are cited in the supplementary materials.
