# Supplementary material for: Flammulina velutipes Mycorrhizae Attenuate High Fat Diet-Induced Lipid Disorder, Oxidative Stress and Inflammation in the Liver and Perirenal Adipose Tissue of Mice
Source: Nutrients. 2022 Sep 16;14(18):3830. doi: 10.3390/nu14183830 (PMC9505303; doi:10.3390/nu14183830)
Supplement: Supplementary file 1 [file nutrients-14-03830-s001.zip › nutrients-1889794-supplementary Table S1-S3.pdf]

Table S1 Primers used in this study.

Target	Primer sequence	No.
Slc27a1	F: 5'-GCTTCAACAGCCGTATCCTC-3’	NM_001357180.1
	R: 3'-TCTTCTTGTTGGTGGCACTG-5'	
CD36	F: 5'-TGCTGGAGCTGTTATTGGTG-3’	NM_001159558.1
	R: 3'-TGGGTTTTGCACATCAAAGA-5'	
FABP1	F: 5'-GCAGAGCCAGGAGAACTTTG-3’	NM_017399.5
	R: 3'-GTCTCCAGTTCGCACTCCTC-5'	
ATGL	F: 5'-ACAGTGTCCCCATTCTCAGG-3’	NM_001163689.1
	R: 3'-TTGGTTCAGTAGGCCATTCC-5'	
HSL	F: 5'-AGACACCAGCCAACGGATAC-3’	NM_001039507.2
	R: 3'-ATCACCCTCGAAGAAGAGCA-5'	
MGLL	F: 5'-CAAGGCCCTCATCTTTGTGT-3’	NM_001166249.1
	R: 3'-ACTTGGAAGTCCGACACCAC-5'	
CPT1α	F: 5'-CCAGGCTACAGTGGGACATT-3'	NM_013495.2
	R: 3'-GAACTTGCCCATGTCCTTGT-5'	
MACD	F: 5'-AGGTTTCAAGATCGCAATGG-3’	NM_007382.5
	R: 3'-CTCCTTGGTGCTCCACTAGC-5'	
SREBP1	F: 5'- CACTCAGCAGCCACCATCTA-3’	NM_001358314.1
	R: 3'-GCTGTCAGCAGCAGTGAGTC-5'	
FASN	F: 5'-TGGGTTCTAGCCAGCAGAGT-3’	NM_007988.3
	R: 3'-ACCACCAGAGACCGTTATGC-5'	
Acaca	F: 5'-GCCTCTTCCTGACAAACGAG-3’	NM_133360.2
	R: 3'-TGACTGCCGAAACATCTCTG-5'	
PGC1α	F: 5'-ATGTGTCGCCTTCTTGCTCT-3’	NM_008904.2
	R: 3'-ATCTACTGCCTGGGGACCTT-5'	
TFAM	F: 5'-CAGGAGGCAAAGGATGATTC-3’	NM_009360.4
	R: 3'-ATGTCTCCGGATCGTTTCAC-5'	
Nrf1	F: 5'-CAACAGGGAAGAAACGGAAA-3’	NM_001164226.1
	R: 3'-GCACCACATTCTCCAAAGGT-5'	
Nrf2	F: 5'-CTCGCTGGAAAAAGAAGTGG -3’	NM_010902.4
	R: 3'-CCGTCCAGGAGTTCAGAGAG-5'	
IL1β	F: 5'-AGTTGCCTTCTTGGGACTGA-3’	NM_008361.4
	R: 3'-TCCACGATTTCCCAGAGAAC-5'	
IL6	F: 5'-AGTTGCCTTCTTGGGACTGA-3’	NM_001314054.1
	R: 3'-CAGAATTGCCATTGCACAAC-5'	
TNFα	F: 5'-CGTCAGCCGATTTGCTATCT-3’	NM_001278601.1
	R: 3'-CGGACTCCGCAAAGTCTAAG-5'	
CCL-2	F: 5'-AGGTCCCTGTCATGCTTCTG-3’	NM_011333.3
	R: 3'-TCTGGACCCATTCCTTCTTG-5'	
TGFβ	F: 5'-TGCTAATGGTGGACCGCAA-3’	NM_011577.2
	R: 3'-CACTGCTTCCCGAATGTCTGA-5'	
IL10	F: 5'-TGTCAAATTCATTCATGGCCT-3’	NM_010548.2
	R: 3'-ATCGATTTCTCCCCTGTGAA-5'	

CD206	F: 5'-CATGGATGTTGATGGCTACTGGAG-3’<br>R: 3'-GTCTGTTCTGACTCTGGACACTTG-5'	NM_008625.2
Fn1	F: 5'-GGAGTGGCACTGTCAACCTC-3’<br>R: 3'-ACTGGATGGGGTGGGAAT-5'	NM_001276413.1
β-actin	F: 5'-AGCCATGTACGTAGCCATCC-3’<br>R: 3'-CTCTCAGCTGTGGTGGTGAA-5'	NM_007393.5

FATP1/Slc27a1: fatty acid transport protein 1; CD36: fatty acid translocase; FABP1: fatty acid-binding protein; ATGL: adipose triglyceride lipase; MGLL: monoacylglycerol lipase; HSL: hormone sensitive lipase; CPT1α: carnitine O-palmitoyltransferase 1; MACD: medium-chain acyl-CoA dehydrogenase; SREBP1: sterol regulatory element-binding protein 1; FASN: fatty acid synthase; Acaca: acetyl-Coenzyme A carboxylase alpha. PGC1α: peroxisome proliferator-activated receptor gamma coactivator 1-alpha; TFAM transcription factor A, mitochondrial; Nrf1: nuclear respiratory factor 1; Nrf2: nuclear respiratory factor 2; TNFα: tumor necrosis factor α; CCL2: C-C motif chemokine 2; TGFβ1: transforming growth factor beta-1; Fn1: fibronectin 1; CD206: macrophage mannose receptor 1; The primers of TGFβ, IL10, Fn1 and CD206 were according to Liu et al.

Reference

Liu Y.; Wang L.; Luo M.; Chen N.; Deng X.; He J.; Zhang L.; Luo P.; Wu J. Inhibition of PAI-1 attenuates perirenal fat inflammation and the associated nephropathy in high-fat diet-induced obese mice. Am. J. Physiol. Endoc. M. 2019, 316, E260-E267.

Table S2 The nutritional composition, harmful substances and heavy metals in FV mycorrhizae.

Items	FV mycorrhizae	Methods
**Nutritional composition**		
CP (%/DM)	16.91 ± 0.12	GB/T 6432-1994
EE(%/DM)	2.34 ± 0.08	GB/T 6433-2006
CF (%/DM)	17.57 ± 0.98	GB/T 6434-2006
Ash (%/DM)	8.19 ± 0.14	GB/T 6438-2007
NFE (%/DM)	54.99 ± 2.35	
Ca (%/DM)	0.60 ± 0.05	GB/T 6436-2002
P (%/DM)	0.70 ± 0.03	GB/T 6437-2002
Fe	320	GB/T 13885-2017
Mn	48	GB/T 13885-2017
Zn	38	GB/T 13885-2017
Cu	9.3	GB/T 13885-2017
Se	0.07	GB/T 13883-2008
Na	560	GB/T 13885-2017
Mg	2800	GB/T 13885-2017
K	34000	GB/T 13885-2017
**Harmful substances**		
AFB1 (μg/kg)	ND	
ZEN (μg/kg)	ND	
DON (mg/kg)	<0.1	
Salmonella	ND	
**Heavy metals (mg/kg)**		
Pb	0.24	GB/T 13080-2018 7.2
Hg	0.01	GB/T 13081-2006 4
Cd	ND	GB/T 13082-2021 8.3.2
As	0.18	GB/T 13079-2006 7

AFB1: Aflatoxin B1; ZEN: Zearalenone; DON: Deoxynivalenol; ND: not detected;

Table S3 Amino acids contents and derivatives in FV mycorrhizae (μmol/g)

Item		FV mycorrhizae
EAAs		
	Thr	2.063 ± 0.055
	Leu	0.768 ± 0.024
	Ile	0.576 ± 0.015
	Met	0.002 ± 0
	Val	1.663 ± 0.024
	Lys	2.952 ± 0.066
	Phe	1.142 ± 0.043
	Trp	0.097 ± 0.002
NEAAs		
	Tyr	3.43 ± 0.067
	Asp	1.281 ± 0.037
	Asn	0.82 ± 0.016
	Pro	0.863 ± 0.016
	Ala	8.968 ± 0.258
	Ser	2.834 ± 0.098
	Gly	1.686 ± 0.083
	Arg	2.941 ± 0.147
	Gln	4.52 ± 0.089
	Glu	6.898 ± 0.174
	Cys	ND
	His	0.159 ± 0.002
	Cystine	0.016 ± 0.003
Derivatives		
	Citrulline	0.049 ± 0.001
	Ornithine	3.805 ± 0.075
	Hydroxyproline	0.131 ± 0.002
	Aminoadipic acid	0.07 ± 0.002
	Creatine	0.016 ± 0.001
	Creatinine	0.001 ± 0
	Choline	65.925 ± 1.719
	Taurine	0.007 ± 0
Biogenic amines		
	Spermidine	ND
	Putrescine	0.057 ± 0.003

EAAs: essential amino acids; NEAAs: non-essential amino acids; ND: not detected.
